# Expression of Concern: LncRNA SRA1 is downregulated in HPV-negative cervical squamous cell carcinoma (CSCC) and regulates cancer cell behaviors

**DOI:** 10.1042/BSR-20191226_EOC

**Published:** 2020-07-02

**Keywords:** cervical squamous cell carcinoma, HPV, lncRNA SRA1, miR-9

The Editorial Office has been made aware of potential issues surrounding the scientific validity of this paper, hence has issued an expression of concern to notify readers whilst the Editorial Office investigates.

